# Physical Diagnosis

**Published:** 1882-08

**Authors:** J. B. Hodgkin


### EDITORIAL, ETC.

---

*Physical Diagnosis.*—The diagnosis of pathological conditions in certain tooth troubles is often obscured by inability on the part of the patient to point out the offending member. The greatest assiduity and acuteness is called for on the part of the dental practitioner to avoid mistakes. In illustration of this. not long since a remarkably careful operator removed a large filling from a superior bicuspid on account of supposed dead pulp, but on drilling beyond the cavity unmistakable signs of vitality were manifested. Yet there was a fistulous opening in the alveolus opposite the apex of the root of the suspected tooth. Careful exploration revealed a necrosed plate of alveolus, the cause not discernable, and with its removal the trouble ceased.

The following method has been helpful in cases where there was manifest trouble but difficult of location. Place the tip of the finger against the side of the tooth most distant from the operator; rap upon the tooth with the end of an excavator. The sense of touch will sometimes enable one to detect looseness in the tooth when ordinary signs fail. It is like pulse diagnosis, requiring cultivation of touch. Something may be done with exastosed roots of teeth in the same way.

There is a good book by Hilton of England, on the "Influence of Rest in Pain,' which contains some practical lessons in diagnosis which are well worth study. The principles he lays down are in many cases applicable to dental practice. It will pay to read this book, and it is a cheap one.

In connection with this subject of diagnosis the writer saw a few days ago what appeared to be a bad abscess, at the root of a lower inscisor. But as the tooth responded to the usual tests for vitality careful examination showed that salivary calculus was the cause of the ulceration.

Occasionally we see the tips of roots of teeth partly protruding, the tooth still firm. To amputate these protruding ends is a neat operation, and often successful, Also amputation of roots of abscessed teeth has proven successful. This if practicable is better than extracting and replacing; with either spray and the dental engine the operation might be brief and comparatively painless.

J. B. HODGKIN.

